# Proteomic profiling of colorectal liver metastases reveals histopathological response-specific molecular signatures of chemotherapy efficacy

**DOI:** 10.1186/s12967-026-07945-1

**Published:** 2026-03-05

**Authors:** Agnes K. Böhm, Lisa M. Skrip, Oliver Klein, Felix Strobl, Jonas K. Wieland, Alexander Arnold, Yijun Zhou, Björn Papke, Christine Sers, Dominik P. Modest, Simon Moosburner, Philipp K. Haber, Felix Krenzien, Nathanael Raschzok, Wenzel Schöning, David Horst, Thomas Malinka, Ingolf Sack, Johann Pratschke, Igor M. Sauer, Karl H. Hillebrandt

**Affiliations:** 1 Department of Surgery, Experimental Surgery, Charité – Universitätsmedizin Berlin, Corporate Member of Freie Universität Berlin and Humboldt-Universität zu Berlin, Augustenburger Platz 1, 13353 Berlin, Germany; 2 Core Facility Imaging Mass Spectrometry, Berlin Institute of Health at Charité – Universitätsmedizin Berlin, 13353 Berlin, Germany; 3 Department of Vascular Surgery, Charité – Universitätsmedizin Berlin, Corporate Member of Freie Universität Berlin and Humboldt-Universität zu Berlin, Hindenburgdamm 30, 12203 Berlin, Germany; 4 Institute of Pathology, Charité – Universitätsmedizin Berlin, Corporate Member of Freie Universität Berlin and Humboldt-Universität zu Berlin, Charitéplatz 1, 10117 Berlin, Germany; 5 Department of Hematology, Oncology, and Cancer Immunology (CVK/CCM), Charité – Universitätsmedizin Berlin, Corporate Member of Freie Universität Berlin and Humboldt- Universität zu Berlin, Charitéplatz 1, 10117 Berlin, Germany; 6 Berlin Institute of Health at Charité – Universitätsmedizin Berlin, BIH Biomedical Innovation Academy, BIH Charité Clinician Scientist Program, Charitéplatz 1, 10117 Berlin, Germany; 7 Department of Radiology, Charité – Universitätsmedizin Berlin, Corporate Member of Freie Universität Berlin and Humboldt- Universität zu Berlin, Charitéplatz 1, 10117 Berlin, Germany; 8 Charité – Universitätsmedizin Berlin, Corporate Member of Freie Universität Berlin and Humboldt Universität zu Berlin, Cluster of Excellence Matters of Activity. Image Space Material funded by the Deutsche Forschungsgemeinschaft (DFG, German Research Foundation) under Germany’s Excellence Strategy – EXC 2025, Berlin, Germany; 9 Department of Surgery Experimental Surgery, Charité – Universitätsmedizin Berlin, Campus Charité Mitte I Campus Virchow Klinikum Augustenburger Platz 1, 13353 Berlin, Germany

**Keywords:** Proteomics, Colorectal liver metastases, Colorectal cancer, Chemotherapy response, Biomarker, Histopathological response

## Abstract

**Background:**

Chemoresistance in treatment of colorectal liver metastases (CRLM) poses a major challenge in preventing disease relapse, with up to 80% of patients developing drug resistance over the course of treatment. Proteomic signatures of responsive vs. non-responsive metastases can provide insights into functional expression patterns, potentially identifying biomarkers for therapy efficacy.

**Methods:**

A total of 33 CRLM tissue samples from 31 patients were subjected to histopathological and proteomic analysis. The patients were included in the study after undergoing preoperative treatment (*n* = 28), including platinum-based chemotherapy (*n* = 19), non-platinum-based chemotherapy (*n* = 8), as well as targeted therapies (*n* = 20), or without preoperative therapy (*n* = 5). Based on Rubbia-Brandt criteria and vital tumor cell percentage, CRLM were categorized to major (MR), partial (PR) and no response (NR) groups. Proteomic analysis was conducted using label-free mass spectrometry (LFQ-MS), followed by clustering according to histological response and distinct treatment regimens.

**Results:**

Proteomic analysis revealed significant differential protein expression of 607 proteins linked to distinct histopathological response types. CRLM responsive to chemotherapy displayed marked enrichment (*p* ≤ 0.01) in pathways associated with immune infiltration, ECM matrix organization, the complement system, and apolipoprotein-associated processes, indicative of distinct stromal and immune invasion patterns with multimodal importance of cell adhesion proteins. In contrast, attenuated expression of proteins enriched in pathways of mitochondrial translation initiation, elongation and termination was detectable.

**Conclusion:**

CRLM exhibited distinct proteomic phenotypes based on their histopathological response to preoperative systemic therapy largely independent of chemotherapy regimens. This proteomic profiling establishes a foundation for identifying critical biomarker profiles by nominating protein markers for major chemotherapy response.

**Supplementary Information:**

The online version contains supplementary material available at 10.1186/s12967-026-07945-1.

## Graphical Abstract

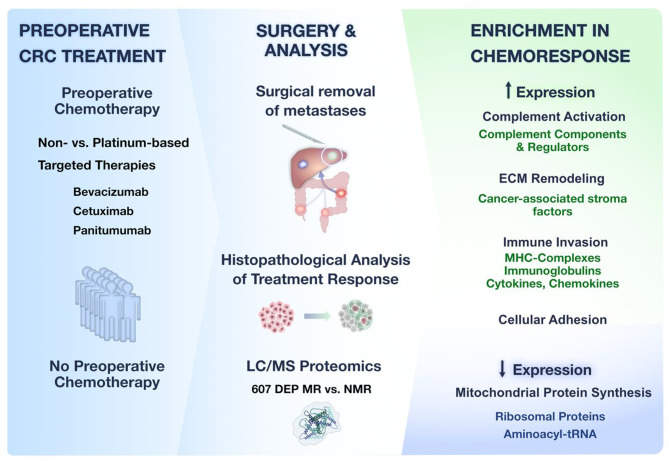

## Introduction

Colorectal liver metastases (CRLM) represent the most prevalent metastatic manifestation of colorectal cancer (CRC), which ranks third most common cancer and second leading cause of cancer-related deaths [1]. In approximately half of cases diagnosed at an advanced stage, about 50% of CRC patients will develop CRLM, with surgical treatment being primarily feasible in around 10 to 20% of patients [2, 3]. Apart from 70% of metastasized colorectal cancer cases with unresectable liver metastases, preoperative chemotherapy can be a viable choice for borderline resectable metastases to be converted to a resectable status, as the definitive removal of CRLM represents the sole curative treatment option [4, 5]. The effectiveness of the first-line therapy constitutes a prime outcome factor, whereby both clinical and molecular markers influence the choice of first-line therapy [6]. A multitude of individual patient-related factors, prior therapeutic approaches and intrinsic tumor properties are taken into account when determining the most appropriate guideline-based therapy regimen. Treatment strategies are continuously evolving, while current first-line therapy typically consists of a doublet or triplet regimen based on 5-fluorouracil (5-FU) and folinic acid, combined with a platinum-based agent such as oxaliplatin (FOLFOX) and/or a topoisomerase inhibitor like irinotecan (FOLFIRI) [7, 8]. When indicated, these regimens are further supplemented with targeted therapies [7, 8]. 

With response rates to first-line therapy range from only 40 to 65%, and further decline following second- or third-line therapy, chemoresistance remains a widely prevalent and critical challenge, affecting up to 80% of patients during the course of treatment [6, 9]. Drug resistance in CRC is driven by both static and dynamic mechanisms – including inherent or acquired genomic alterations, dysregulated gene expression, changes in drug processing, and altered cancer metabolism – posing a continuous challenge to effective treatment [10]. Secondary resistance typically develops within 3 to 12 months after initial therapeutic response, driven by molecular alterations and persistent changes in gene expression induced by chemotherapy [11, 12]. Given that metastatic recurrence affects up to 70% of patients, identifying specific molecular patterns linked to treatment response may prove decisive for improving outcomes [9]. The effective and precise targeting of CRLM, particularly in cases resistant to chemotherapy, may require identifying their unique molecular signatures. Molecular profiling could enable earlier, personalized therapeutic decisions by tailoring strategies to individual risk profiles and anticipated clinical benefit. Proteomic analyses offer a powerful tool to provide insight into the immediate compositional and functional state, including adaptive mechanisms such as drug resistance, which may rely more heavily on protein interactions than on gene expression alone [13]. Given the substantial molecular heterogeneity of CRC and the limited characterization of the molecular features of CRLM, our aim is to contribute to broadening this understanding by specifically analyzing CRLM in relation to treatment efficacy [14]. In our study, we investigated the proteomic composition of CRLM in relation to administered chemotherapy regimen and chemotherapy efficacy, nominating potential robust biomarker candidates for detection of major chemotherapy efficacy.

## Methods

### Acquisition and histopathological analysis of samples

Between October 2022 and May 2023, a total of 31 patients who underwent surgical resection at the Department of Surgery, Campus Charité Mitte and Campus Virchow Klinikum, Charité – Universitätsmedizin Berlin, were included in the study. All patients provided written informed consent for tissue sampling prior to surgery. The study was approved by the Charité ethics committee (approval numbers: EA1/214/19 and EA4/132/22) and was conducted in accordance with the principles of the Declaration of Helsinki.

**Fig. 1:**
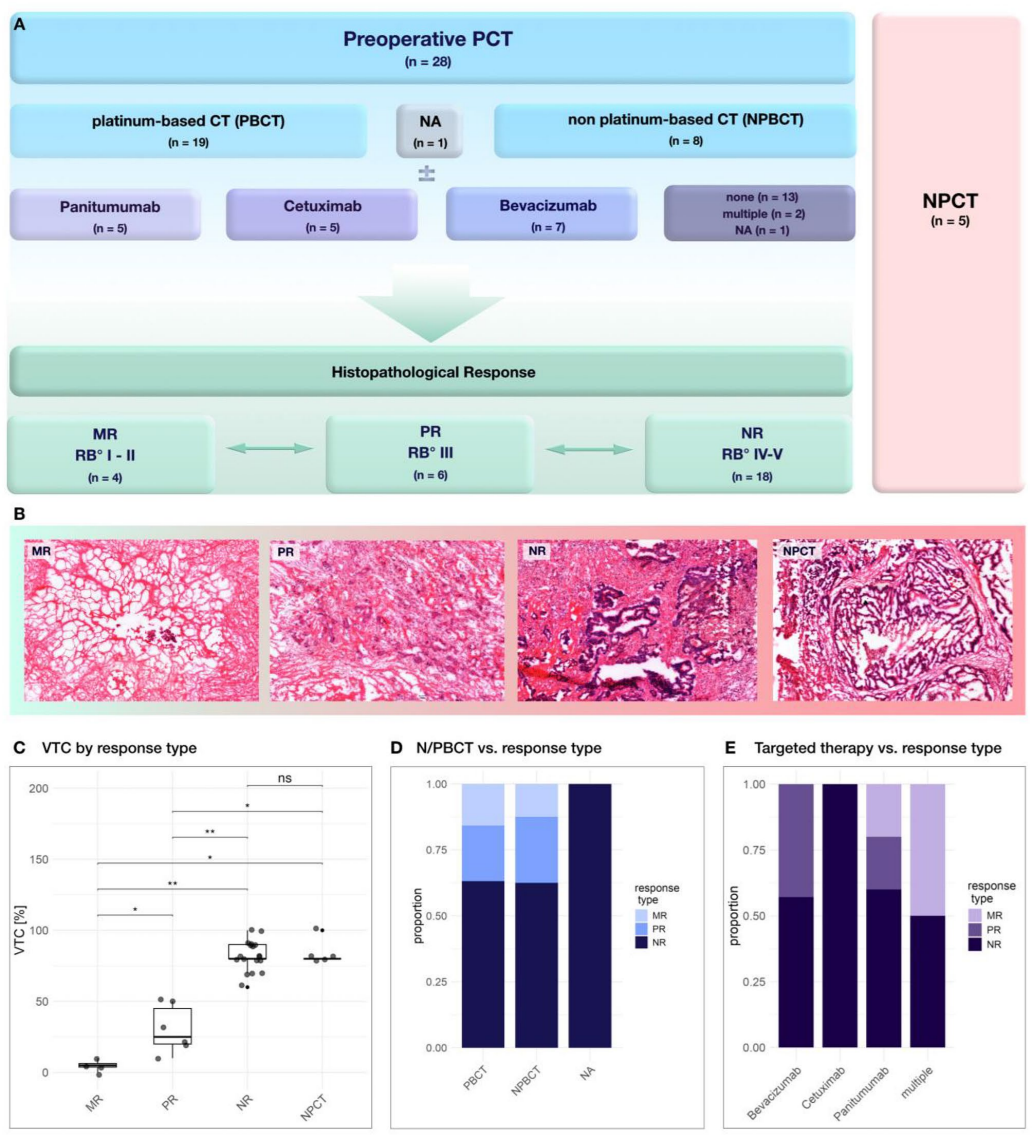
**(A)** Study design: Patients receiving preoperative chemotherapy (PCT) were compared with patients receiving no preoperative chemotherapy (NPCT) treatment regimen. Histopathologic response was assessed by Rubbia-Brandt (RB) grading and residual vital tumor cell (VTC) count (in %). **(B)** Representative histopathologic results for each group (major response – MR, partial response – PR, no response – NR, and NPCT), with HE staining. A gradual decrease of tumor cellularity (dark purple), concomitant with a relative increase of fibrosis from non-responsive tissue samples and no-pretreated tumor samples (NR, NPCT) to responsive tissue samples (MR, PR). **(C)** VTC by histopathological response, statistical analysis by pairwise Wilcoxon rank test with significance * *p* ≤ 0.05, ** *p* ≤ 0.01. Distribution of **(D)** PBCT vs. NPBCT and **(E)** targeted therapy regimes across histopathologic subtypes. The PBCT vs. NPBCT groups showed comparable distributions of chemotherapy response. Among targeted therapies, cetuximab recipients showed no response to chemotherapy, whereas panitumumab recipients demonstrated the best response

Surgically resected tissue samples were gently rinsed in PBS buffer and stored at − 20 °C until further use. For assessment of histopathological response, metastases were categorized by a board-certified pathologist based on the area of necrosis and fibrosis (ANF) according to the Rubbia-Brandt (RB) classification, in conjunction with the percentage of vital tumor cells (VTC) [15]. A major response (MR) was defined as Rubbia-Brandt grade I/II with ANF ≤ 25 and a VTC ≤ 10%. Partial response (PR) corresponded to RB grade III with an ANF between 26 and 75% and a VTC within the range of 10–50%. No response (NR) was defined as RB grade IV/V with an ANF of ≥ 75% and a VTC of ≥ 50% (Fig. 1B, C).

### Experimental design and statistical rationale

#### Methodology of proteomic analysis

Protein identification was performed using a nano-liquid chromatography electrospray ionization tandem mass spectrometry (LC-MS) approach as previously described by *Wisniewksi et al.* (2009) [16]. All samples were lyophilized, homogenized, and prepared for LC/MS using the *Filter-Aided Sample Preparation* (FASP) method, involving the injection of 4 µl of eluate into a *nanoUHPLC* system (Dionex UltiMate 3000, Thermo Fisher Scientific, Waltham, MA, USA) coupled with an timsTOF HT flex mass spectrometer (Bruker Daltonics) equipped with a CaptiveSpray nano-electrospray ion source 2 (timsTOF flex HT, Bruker Daltonic GmbH, Bremen, Germany) [16]. Spectra libraries were created with pool samples by data in data dependent analyses (DDA), as further described in Supplementary Data 1. Parallel accumulation-serial fragmentation combined with data-independent acquisition (diaPASEF) was performed [17]. Spectra library was used to search against the acquired diaPASEF raw data files via PEAKS studio proteomics search engine (Version 11.5 Bioinformatics Solutions, Waterloo, Canada). Label-free quantification (LFQ) with PEAKS Q was used, whereby PEAKS was allowed to autodetect the reference sample and automatically align the sample runs. To facilitate the export of complete results, the protein significance filter was set to 0, the protein fold change filter to 1, and unique peptide filter to 1 in the export settings. All primary proteomic data was uploaded to the *ProteomeXchange Consortium* via the *PRIDE partner repository* (PXD065812) [18]. 

#### Selection criteria and database search

A qualitative analysis first aimed to ensure robustness by establishing presence criteria for subgroup analysis: (a) unique-peptide-to-peptide ratio ≥ 0.2, (b) unique peptide count ≥ 2, and (c) coverage ≥ 5 in at least 75% of patients per subgroup. Proteins were included in downstream analysis if classified as “present” in at least one of the compared groups. The subgrouping was based on histopathological response (MR, PR, NR), platinum- (PBCT) vs. non-platinum-based chemotherapy (NPBCT), and targeted therapy (*Bevacizumab*,* Cetuximab*,* Panitumumab*). Multiple databases were utilized for interpretation of proteomic output: after identification of protein IDs by UniProt, online databases Reactome and StringDB were used for enrichment analysis of differentially expressed proteins (DEP) [19–21]. Matrisome analysis via *MatrisomeDB* was conducted for all proteins in the histopathological response subset [22]. 

#### Statistical analysis

The proteomic data was processed and statistically analyzed in R Studio (Version 2023.12.0), and all graphical output was produced via multiple packages included in Supplementary Data 2. The quantitative analysis employed the use of LFQ data normalized on the peptide level for the statistical evaluation of subgroup differences. Multiple group comparisons were conducted using the Kruskal-Wallis test with Dunn’s post-hoc test, while Welch’s t-test followed by the Wilcoxon rank-sum test was used for two-group comparisons. FDR-correction by the Benjamini-Hochberg method was applied for all differential expression analyses, with significance set at q ≤ 0.05 and fold change (FC) ≤ 0.5 or ≥ 2. To enable FC calculation, absent proteins were assigned a small pseudo count. Only protein expressions that were statistically significant were visualized in volcano plots for response group analysis, while all proteins were visualized comparing NPCT vs. NR and NPBCT vs. PBCT. Principal component analysis (PCA) was performed on the mean protein area matrix across response groups (MR, PR, NR) using the *prcomp* function with z-score scaling, with missing values were imputed as zero prior to analysis. For volcano plots and heatmaps, proteins with BH-corrected FDR < 0.05 and significant fold changes were visualized, with a red line marking the p-value threshold (*p* < 0.05). In volcano plots, green and blue lines denote fold-change cutoffs (FC < 0.5 and FC > 2, respectively). For heatmaps, row-wise z-scores were calculated and heatmaps were visualized with the *pheatmap* function and Euclidean clustering. Pearson’s Chi-squared test was employed to assess categorical patient characteristics, and the Mann-Whitney U test was used for additional two-group analysis.

## Results

### Overview of the study

A total of 33 metastases from 31 patients were included in the study. Two patients underwent two-staged resection of two metastases, with a mean interval of 41 days between surgeries. While a total of 28 metastases were exposed to preoperative chemotherapy (PCT), five metastases were classified as the non-pretreated (*no preoperative chemotherapy*, NPCT) subset. Of the metastases included in the study, 13 were treated with a single chemotherapy regimen. Eleven metastases were resected after the patient had transitioned to a second-line regimen. Two metastases were operated after a total of three regimens of chemotherapy, and one metastasis was exposed to de-escalation and re-escalation in four steps. Therapy data for one metastasis was unavailable. PBCT was administered in 19 cases, while eight metastases were treated with NPBCT. EGFR-inhibitors were applied in 10 cases, with *Panitumumab* or *Cetuximab* administered in five cases each. Seven metastases were treated with anti-VEGF therapy with *Bevacizumab*. Detailed therapy regimens are shown in Fig. 1A; Table 1. Among the histopathologic response types, patient characteristics did not differ with respect to age, sex and tumor origin (Table 1). No significant differences in the response type distribution were observed with respect to chemotherapy regimen, neither antibody therapy nor for PCT vs. NPBCT (Fig. 1D, E; Table 2).

**Table 1:** Cohort characteristics. CUP – cancer of unknown primary

Characteristic	Overall *N* = 33	MR *N* = 4	PR *N* = 6	NR *N* = 18	NPCT *N* = 5	*p*-value
sex						0.6
*female*	12 (36%)	1 (25%)	1 (17%)	8 (44%)	2 (40%)	
*male*	21 (64%)	3 (75%)	5 (83%)	10 (56%)	3 (60%)	
age (mean ± SD)	62 (11)	54 (12)	64 (13)	62 (11)	69 (8)	0.2
primary cancer						0.5
*colon*	8 (24%)	1 (25%)	3 (50%)	3 (17%)	1 (20%)	
*sigma*	18 (55%)	1 (25%)	2 (33%)	12 (67%)	3 (60%)	
*rectum*	6 (18%)	2 (50%)	1 (17%)	2 (11%)	1 (20%)	
*CUP*	1 (3.0%)	0 (0%)	0 (0%)	1 (5.6%)	0 (0%)	
synchronicity						0.7
*metachronous*	13 (39%)	2 (50%)	2 (33%)	6 (33%)	3 (60%)	
*synchronous*	20 (61%)	2 (50%)	4 (67%)	12 (67%)	2 (40%)	
VTC (mean ± SD)	64 (32)	5 (4)	30 (17)	82 (11)	84 (9)	< 0.001
UICC						0.12
*I*	1 (3.0%)	0 (0%)	0 (0%)	0 (0%)	1 (20%)	
*II*	2 (6.1%)	1 (25%)	0 (0%)	0 (0%)	1 (20%)	
*III*	7 (21%)	0 (0%)	2 (33%)	5 (28%)	0 (0%)	
*IV*	23 (70%)	3 (75%)	4 (67%)	13 (72%)	3 (60%)	

SD – standard deviation. VTC – vital tumor cell percentage

**Table 2:** Cohort – treatment and chemotherapy response

Characteristic	Overall *N* = 33	MR *N* = 4	PR *N* = 6	NR *N* = 18	*p*-value
VTC (mean ± SDT)	60 (33)	5 (4)	30 (17)	82 (11)	< 0.001
Radiological Response (RECIST 1.1)					0.075
*PD*	3 (11%)	0 (0%)	0 (0%)	3 (17%)	
*PR*	9 (32%)	4 (100%)	1 (17%)	4 (22%)	
*STD*	14 (50%)	0 (0%)	5 (83%)	9 (50%)	
*Unknown*	2 (7.1%)	0 (0%)	0 (0%)	2 (11%)	
Platinum-based CT					> 0.9
*non-platinum based*	8 (30%)	1 (25%)	2 (33%)	5 (29%)	
*platinum-based*	19 (70%)	3 (75%)	4 (67%)	12 (71%)	
*Unknown*	1	0	0	1	
Therapy Regimen					0.8
*Combined (CT + AB)*	19 (68%)	2 (50%)	4 (67%)	13 (72%)	
*CT*	9 (32%)	2 (50%)	2 (33%)	5 (28%)	
CT Cycles					0.6
*> 6*	11 (55%)	1 (33%)	2 (40%)	8 (67%)	
*≤ 6*	9 (45%)	2 (67%)	3 (60%)	4 (33%)	
*Unknown*	8	1	1	6	
Antibody Therapy					0.3
*Bevacizumab*	7 (26%)	0 (0%)	3 (50%)	4 (24%)	
*Cetuximab*	5 (19%)	0 (0%)	0 (0%)	5 (29%)	
*Panitumumab*	5 (19%)	1 (25%)	1 (17%)	3 (18%)	
*multiple*	2	1	0	1	
*none received*	8	2	2	4	
*Unknown*	1	0	0	1	
RAS-Mutation					0.4
*mutation*	8 (40%)	0 (0%)	3 (75%)	5 (36%)	
*wildtype*	12 (60%)	2 (100%)	1 (25%)	9 (64%)	
*Unknown*	8	2	2	4	

CT – chemotherapy. PD – progressive disease. PR – partial response. STD – stable disease. SD – standard deviation. VTC – vital tumor cell percentage

### Main differential protein expression patterns correlate with histopathological response, independent of chemotherapy regimen

**Fig. 2:**
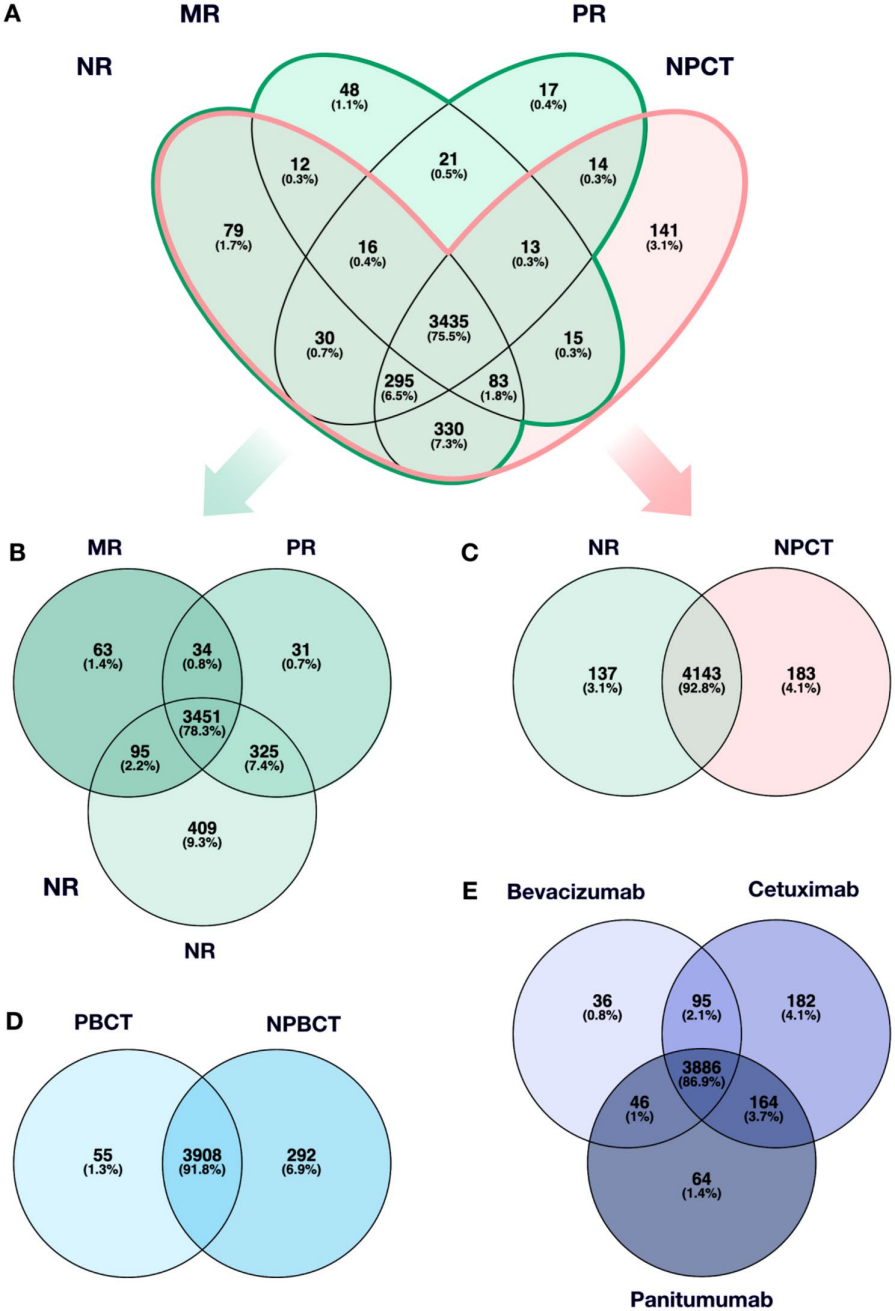
All identified proteins between different subsets, including **(A1)** histopathological response type groups with no-pretreated subset, **(A2)** histopathological subsets exclusively, and **(A3)** NR in comparison to NPCT. **(B)** Common and uniquely present proteins across targeted therapy groups. A large proportion of 87% of proteins was commonly present in all groups, while no DEP were identified. **(C)** PBCT and NPBCT exhibited a predominantly similar protein profile, while differential protein expression remained insignificant

**Fig. 3:**
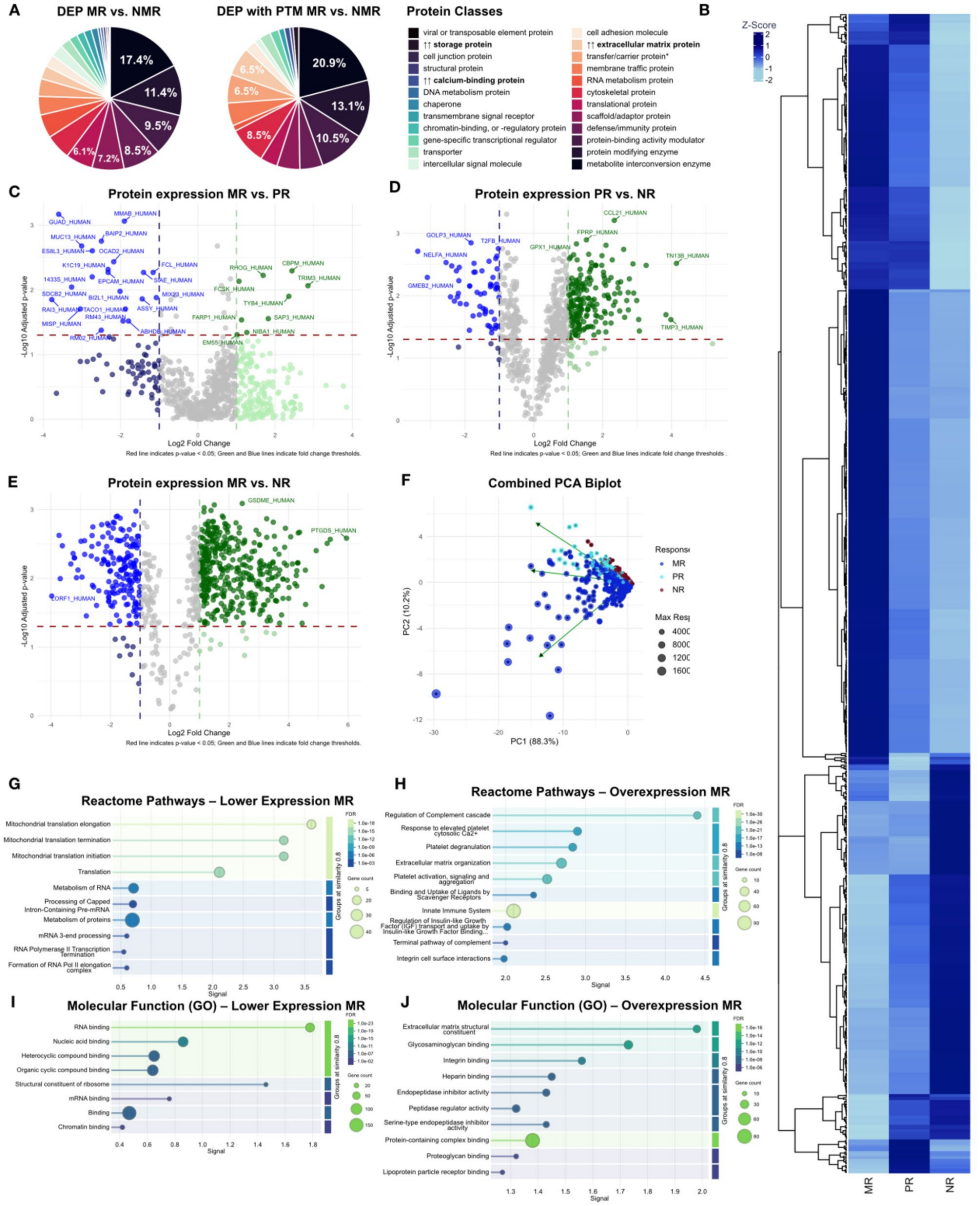
Proteomic analysis of DEP across response types. **(A)** Relative abundance of protein classes represented in DEP (*p* < 0.05) between major (MR) and non-major response (NMR). In relation to overall presence, top three overrepresented post-translational modifications (PTM) were detected in ECM, calcium-binding and storage proteins (upward arrows). **(B)** Heatmap of all DEP (adjusted p value (BH) < 0.05) between groups with row-wise hierarchical clustering. Pairwise group comparisons of **(C)** MR vs. PR, **(D)** PR vs. NR, and **(E)** MR vs. NR. Red line indicates p-value < 0.05; Green and Blue lines indicate fold change thresholds (FC < 0.5, FC > 2). **(D)** PC1 accounts for 88.3% of variance between response groups, with PC2 explaining 10.2%. While PR and NR exhibit similar patterns (green arrows above 0), MR differs in its main components. Attenuated protein expression in MR shows enrichment in **(G)** pathways of mitochondrial translation initiation, elongation and termination, **(I)** mainly of RNA binding proteins. Overexpressed proteins in MR are enriched in **(H)** regulatory processes of the complement cascade and platelet degranulation as well as ECM organization and immune-related pathways. A predominant proportion of enriched protein **(J)** are structural components of the ECM but are also involved in various signaling processes including integrin binding

The initial proteomic qualitative output comprised data on 7,221 proteins, of which LFQ yielded 5,865 proteins (Supplementary Data 3, 4). Across the three response types, qualitative analysis after implementation of quality and universality criteria identified 4,408 proteins, with 3,451 proteins commonly present in all response groups (Fig. 2, A1, A2). NR had the highest number of unique proteins, totaling 409, with a greater overlap shared with PR than with MR. The control subset NPCT (*n* = 4,326; unique *n* = 183) and NR (*n* = 4,280; unique *n* = 137) shared 92.8% (*n* = 4,143) of identified proteins, with no significant differences in protein expression between them (Fig. 2, A3). While the downstream therapy efficacy in the untreated metastases cannot be estimated, the fact that these metastases exhibit a similar proteomic profile to the unresponsive metastases implies that the proteomic changes within major responsive metastases reflect a genuine response to chemotherapy. Consequently, our analysis was conducted from the perspective of the MR subset. Differential expression analysis revealed that significant variation occurred exclusively within histopathological response subtypes, identifying a total of 607 differentially expressed proteins (Supplementary Data 5, Fig. 3, B). A total of 399 proteins exhibited overexpression in MR compared to both PR and NR (*n* = 395 relative to NR, *n* = 9 relative to PR, with an overlap of *n* = 5). Conversely, 177 proteins displayed reduced expression in MR relative to both PR and NR (*n* = 171 compared to NR, *n* = 21 compared to PR, with an overlap of *n* = 15 (Fig. 3A1–3, Supplementary Data 5). PCA revealed a shared proteomic signature across all three response groups, with the most prominent variation captured by PC1 (88.3%). More subtle distinctions between MR and the other groups were observed on PC2 (10.2%; Fig. 3, A4). Individual proteomic PCA plots are provided in Supplementary Data 6. Subgroup analyses of singular regimens of targeted therapy (*Bevacizumab* vs. *Cetuximab* vs. *Panitumumab*, *n* = 4,473 proteins) and N/PBCT (*n* = 4,255 proteins) revealed no significant proteomic differences (Fig. 2, B, C).

### Responsive metastases show pronounced remodeling of the tumor microenvironment

Enrichment analysis of differentially expressed MR proteins identified 34 highly enriched pathways (FDR p-value ≤ 0.01), with a total of 209 proteins (Supplementary data 8). The most significantly enriched pathways (*p* ≤ 0.001) include regulation of the complement cascade (31/135), ECM organization (46/321), response to elevated platelet cytosolic Ca^2+^(30/133) and innate immune system (96/1187; Fig. 3D1, D2). Most enriched pathways entailed complement-related processes. Elevated complement activation shapes the TME across multiple tumor types creating a high tumor-associated inflammation, whereby the interactions highly complex, with pronounced context-duality [23]. Complement activation can effect either pro-tumorigenic or anti-tumorigenic effects depending on the context and activation pathway [23–26]. Recent evidence suggests that complement, coagulation, and cell adhesion proteins are generally enriched in CRLM in comparison to their CRC primaries [27]. Notably, complement-mediated inflammation is a prevalent feature of the mesenchymal consensus molecular subtype 4 (CMS4) of CRC. CMS4 is further characterized by enhanced stromal and immune pan-cancer invasion, increased EMT, increased TGFB signaling, and activation of matrix remodeling – pathways that are collectively enriched in the MR subset (Supplementary Data 9) [23, 28, 29]. The identified CMS4 markers predominantly represent matrisomal proteins of the tumor microenvironment, including biglycan, collagens (COL3A1, COL6A3, COL14A1), and complement components (SERPING1, C1QB) for the stromal type, and immune markers leukocyte adhesion molecules (VCAM1), and immune cell markers (ISLR2, SDF1). Of the stromal markers, 54.5% (*n* = 6) are matrisomal proteins, with 36.4% belonging to the core matrisome and 18.2% to the matrisome-associated compound. Among immune markers, 94.7% were non-matrisomal cell surface proteins, with TGFB1 as the sole secreted matrisomal factor. In contrast, CMS4 markers including ZEB1, FRMD6, KER, CDX2 and HTR2B are primarily epithelial and tumor cell markers, including cytoskeletal and transcription factors expressed in tumor epithelium rather than stroma [30]. 

**Fig. 4:**
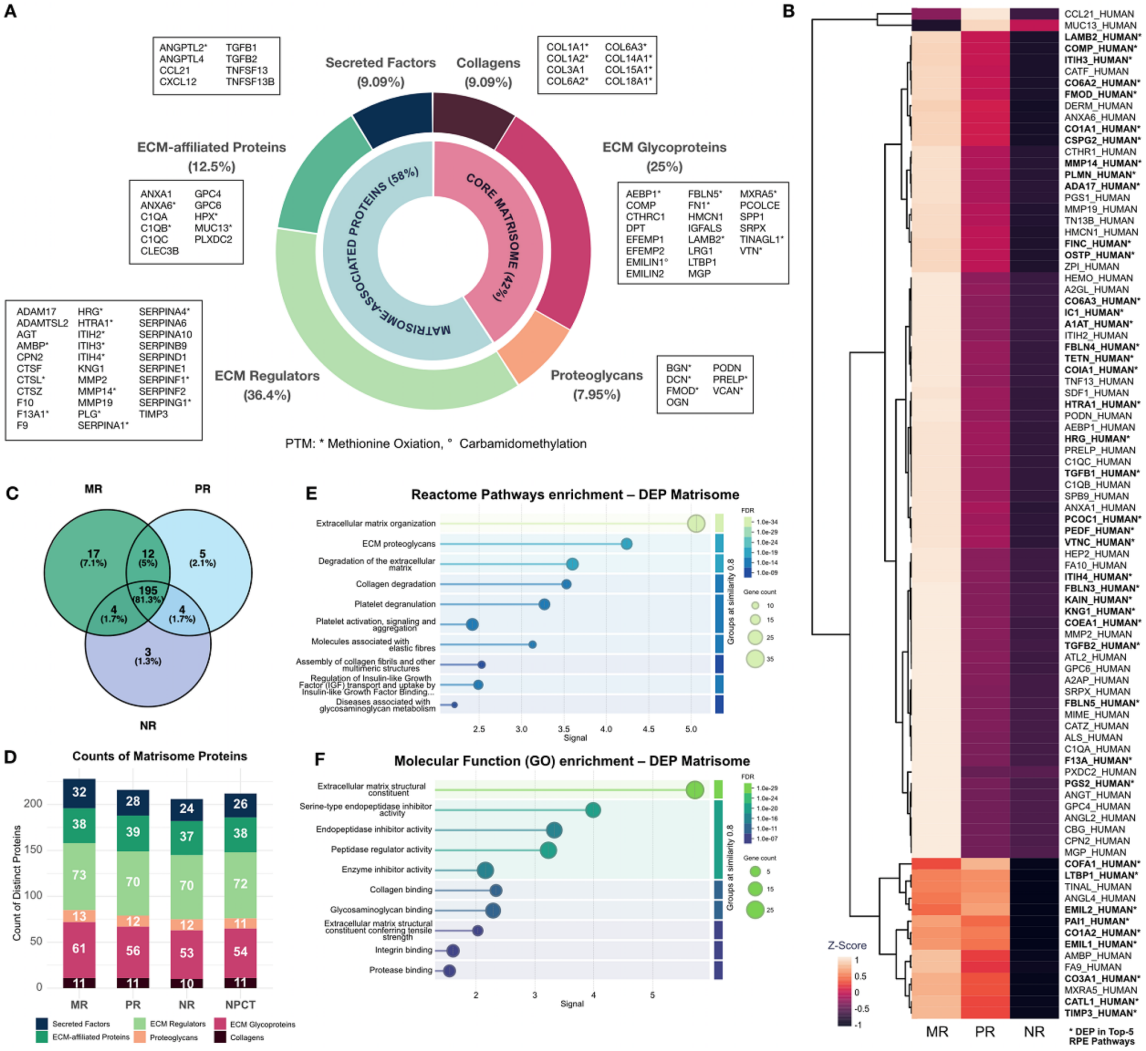
Matrisome analysis of histopathologically distinct metastases. **(A)** Composition of ECM across all histopathological response types (MR, PR, NR), indicated with the underlying gene names. A total of 91 matrisomal proteins are distributed to 37 core matrisomal and 54 matrisome-associated proteins. Of DEPs, PTM primarily affect collagens (87.5%) and proteoglycans (71.4%), followed by ECM-affiliated proteins (45.5%) and ECM regulators (43.8%). **(B)** Relative proportions of matrisomal DEP (adjusted p value (BH) < 0.05) between histopathologic subtypes. The main differentiation pattern relates to upregulation of ECM proteins in MR in comparison to PR and NR. DEP implemented in the top 5 enriched Reactome Pathways are highlighted in bold letters with *. **(C)** Beyond significant differential expression, a total of 81.3% of matrisomal proteins are conserved across histopathologic subtypes, with MR showing the highest proportion of uniquely preserved proteins (*n* = 17). **(D)** Absolute number of preserved protein types relating to matrisome categories. MR preserves the highest number of distinct ECM glycoproteins, ECM regulators and secreted factors. **(E)** Matrisomal DEP are enriched in reactome pathways of ECM remodeling and hemostatic processes and mainly entail ECM structural components, beyond a diverse pattern of enzyme regulatory activity. **(F)** Beyond the predominant structural components forming the ECM network, an enrichment of proteins with enzymatic activity was observed, indicating ongoing ECM remodeling

The histopathologically confirmed stroma-rich architecture of responsive metastases was proteomically reflected in the presence of CRC stroma-associated markers including CSPG-2 and PAI-1 (Fig. 4, A) [5, 25, 31]. Across all histopathological subtypes, a great overlap of 81.3% preserved matrisome proteins was detected, with significantly differential expression in 36.2% (Fig. 4, C, D). Responsive metastases exhibited dominant proportions of differentially expressed matrisomal proteins with overexpression of 81 proteins, with solely one protein being attenuated in responsive metastases (Fig. 4, B***)***. Expression patterns of several markers indicated EMT in responsive metastases. Whereas epithelial markers like Ep-CAM and the less specific Mucin-13 were downregulated in MR metastases [32], markers for epithelial-to-mesenchymal transition (EMT) like the pan-mesenchymal marker vimentin, as well as Cavin-1, COL6A1, COL6A2, COL6A3, LAMB2, MMP-2 and PG-S2 were markedly upregulated [5, 33–35]. ECM remodeling constitutes a dynamic key characteristic both within tumorigenesis, as in epithelial-mesenchymal transition, and cancer responsiveness (Fig. 4, E) [34]. Proteins engaging in ECM remodeling including ECM-degradation proteins of the matrix metalloproteinase family (MMP-2, MMP-14, MMP-19) and inhibitory proteins like TIMP-3 showed elevated expression in MR metastases. The overexpression of the cancer-associated-fibroblast-specific marker FAP and fibroblast-associated PDGFRB, general CAF markers like SDF-1 and PG-S2, as well as mesenchymal vimentin implies CAF presence [5, 25, 33, 36]. CAFs comprise a predominant cellular entity in the microenvironment of CRLM, which along with myofibroblasts predominantly govern ECM remodeling and deposition, thus influencing therapy efficacy [32, 37]. Specifically, markers of two subtypes were enriched in MR metastases, including the general ECM-CAF subtype (12/30), characterized by the fibroblast activation marker prolyl endopeptidase FAP, and a specific subcategory of ECM-CAF, the complement-secreting CAF (10/30), which are associated with complement proteins C3 and C7 (Supplementary Data 7) [25]. 

Post-translational modifications (PTM) were detected in a total of 186 proteins, comprising 183 with methionine oxidation and 3 with carbamidomethylation, of which 40 were matrisomal proteins. Across protein classes, PTMs were most enriched in storage proteins (267% relative proportion), extracellular matrix (ECM) proteins (139%), and calcium-binding proteins (120%) (Fig. 3A**).** Among the differentially expressed matrisomal proteins, methionine oxidation was the predominant PTM, affecting nearly all collagens (87.5%) and a substantial proportion of proteoglycans (71.4%) (Fig. 4A).

### Immune infiltrative patterns indicate cellular diversity in response metastases

**Fig. 5:**
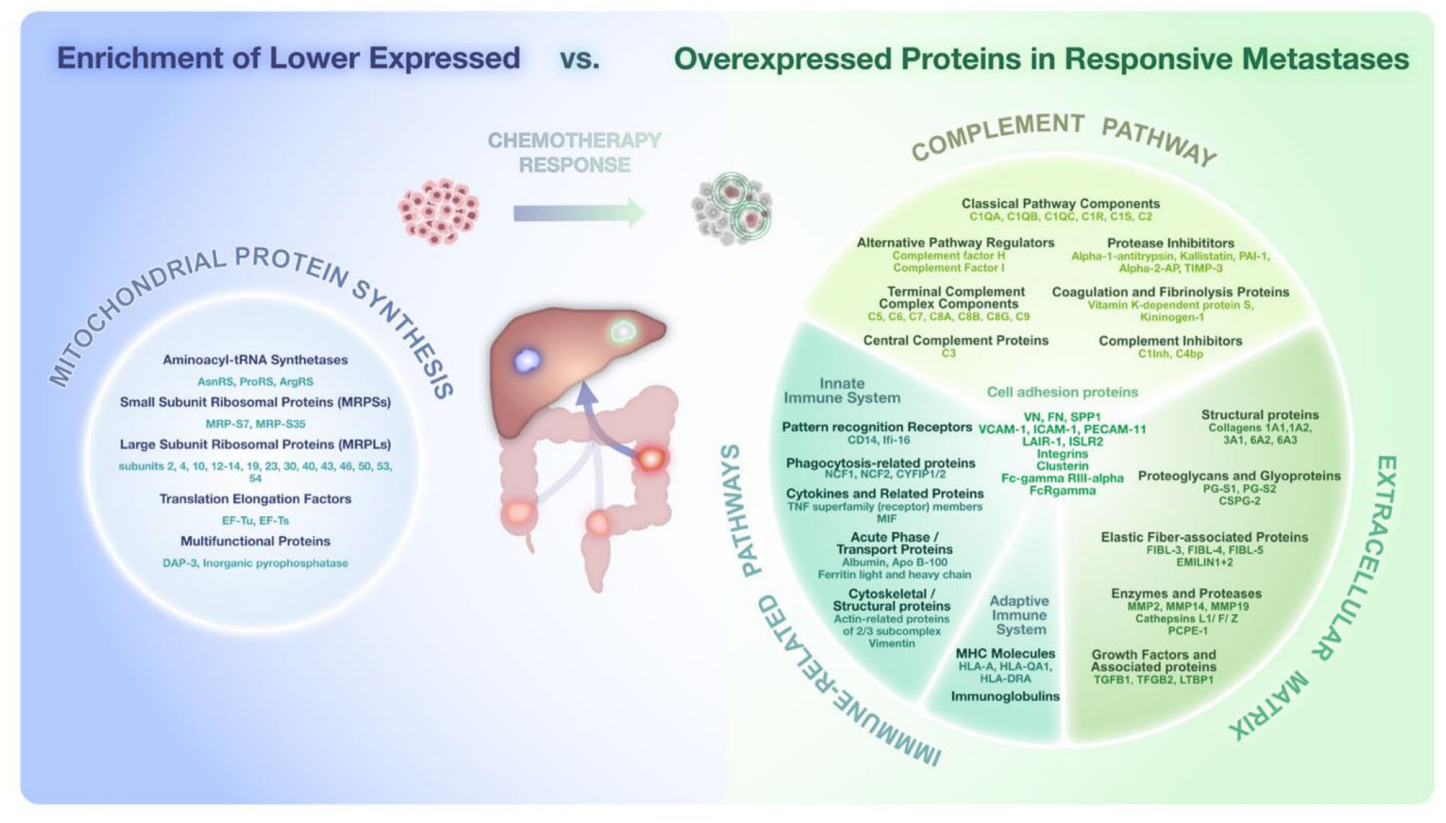
Proteomic enrichment of proteins with attenuated expression (left side) vs. proteins with elevated expression (right side) in CT-responsive metastases with MR

The overexpression of numerous cell adhesion proteins along with other proteins involved in leukocyte recruitment like EC-SOD, inflammatory cytokines and chemokines, and overexpression of TGFB suggest enhanced immune cell signaling, increased stromal interaction, and EMT (Fig. 5) [5, 33, 38–41]. Multiple components of the heterodimer transmembrane receptor family of integrins including alpha-5, alpha-V, alpha-X, and beta-2, which are proposed prognostic markers of CRC, were upregulated in MR [42, 43]. As mediators of inter- and intracellular signaling, integrins and other cell adhesion molecules like LAIR-1, have been implicated in metastatic cascades CRC cell settlement in the hepatic niche, yet exhibit context-dependent duality [42, 43]. While contributing to cancer cell survival and dissemination, adhesion proteins like ICAM-1 and VCAM-1 can enhance anti-tumoral immunity by facilitating immune cell trafficking, cytotoxic lymphocyte adhesion, CAF regulation and drug distribution within established tumor microenvironments [44, 45]. Several CD receptors including the monocyte and macrophage marker CD14 alongside CD18, CD11B, CD206/MRC, CD82, CD74 and MIF, showed elevated expression in MR, indicating profound stromal and immune invasion [46]. The overexpression of some of ApoC-I and ApoE, alongside macrophage ligands such as FLAP, ECM remodeling markers like MMP-2 and MMP-9, and macrophage-associated targets like FN indicates macrophage presence [32]. Elevated levels of complement-associated factors, specifically C1QC, and along with Galectin-3, could support the presence of lipid-associated macrophages that are prevalent in both treated and untreated liver metastases [5, 47]. While the overexpression of SPP1 could be indicative of SPP1 + tumor-associated macrophages, no specific cellular annotation is possible in the bulk proteomic context [32]. Furthermore, MR metastases showed increased levels of immunoglobulins and immunoglobulin receptors, as well as HLA proteins including HLA-A, HLA-DQA1, and HLA-DRA, consistent with an enhanced activation within the immune microenvironment, which can be triggered by chemotherapeutic intervention [5]. Inhering multimodal importance, some of these marker proteins are also associated with distinct CAF-populations, such as MHC-II proteins like CD74 and HLA-DRA, which are abundantly expressed by antigen-presenting CAFs [48]. B cells are generally less prominent in CRLM that in their primaries, and the absence of CD19 as a canonical B cell marker suggests solely low abundance of B cells [5]. Consequently, the exalted presence of immunoglobulins may derive from plasma cells or represent residual protein from prior immune activity. Overall, while the overexpression of protein patterns linked to marked immune infiltration does not allow for the identification of distinct, specific cell types, it instead highlights broad alterations in the immune environment within responsive metastases.

### Responsive metastases exhibit attenuated ribosomal protein biosynthesis

Lower abundant MR proteins were significantly enriched (*p* ≤ 0.01) in five pathways with a total of 24 proteins revolving around mitochondrial translation initiation, elongation and translation (Fig. 3, C1, C2, Supplementary Data 10). The predominant proportion of lower abundant DEP is made up by mitochondrial ribosomal proteins, which along with mitochondrial rRNA assemble into the two subunits of the mitochondrial ribosome, comprising up to 50 individual mitochondrial ribosomal proteins [49, 50]. Besides constituents of the ribosomal complex, associated factors including mitochondrial elongation factors Ts and Tu as well as aminoacyl-tRNA synthetases like ProRS, ArgRS and AsnRS exhibited low expression levels in CT-responsive metastases. On the contrary, cytosolic TrpRS and SerRS showed amplified expression in MR metastases (Fig. 5).

## Discussion

This study employed LC/MS proteomics to investigate global changes in CRLM that show major histopathological response following chemotherapeutic treatment. Given the multitude of factors influencing proteomic composition in CRC and CRLM – including synchronicity, systemic therapies, and primary tumor localization – we adopted an approach to outline common protein expression patterns that could serve as potential biomarkers for predicting chemotherapeutic efficacy across diverse treatment regimens [27, 51, 52]. Using a bulk-proteomic approach with strict criteria for protein detection, we detected robust differential expression of 607 proteins across histopathological response types according to Rubbia-Brandt, largely independent of neoadjuvant chemotherapy treatments including platinum- versus non-platinum chemotherapy or targeted antibody use, none of which significantly altered the proteomic landscape. The absence of significant proteomic differences between non-responders (NR) and non-pretreated controls indicated genuine protein expression changes due to effective chemotherapy response.

As cancer cells experience ongoing evolutionary stress, elevated mitochondrial protein biosynthesis constitutes an active adaptive mechanism to meet the heightened metabolic demands required for their immense adaptability [53, 54]. An elevated state of mitochondrial ribosomal protein synthesis often correlates with increased invasiveness and cellular plasticity, such as required during epithelial-mesenchymal transition (EMT) [53, 54]. Chemotherapy exposure can alter the CRC cells’ energy metabolism, affecting a shift from glycolysis to oxidative phosphorylation, concurrent with an increase in mitochondrial biomass – a phenomenon associated with chemoresistance [55]. The upregulation of multiple ribosomal proteins in CRC has been associated with distinct prognostic outcomes, and similar ribosome gene signatures have been shown to hold prognostic relevance in breast cancer circulating tumor cells, implying that the signatures identified here may have broader applicability [54, 56, 57]. 

Unique expression patterns of aminoacyl-tRNA synthetases have been described in various cancer entities, with point mutations of IARS2, KARS1, and MARS associated with CRC [58]. In chemotherapy-responsive metastases, expression of mitochondrial ARSs such as ProRS, ArgRS, and AsnRS was attenuated, whereas cytosolic SerRS and TrpRS were overexpressed in MR metastases. Lower expression levels of mitochondrial aminoacyl-tRNA synthetases, which serve as a key interface between the proteome and metabolome, indicate diminished core metabolism in responsive metastases, consistent with a reduction in viable tumor cells [50, 58]. Overexpression of these cytosolic ARSs has been associated with protective effects in CRC via Wnt pathway suppression, VEGF-mediated angiogenesis inhibition, and immunomodulation through macrophage activation [58, 59]. 

In chemotherapy-responsive metastases, the most pronounced differential expression patterns were associated with complement-mediated pathways, stromal and immune infiltration, and processes governed by the ECM, indicating profound tumor microenvironment remodeling. The relatively high proportion of mainly methionine oxidation as predominant post-translational modification observed in extracellular matrix proteins, calcium-binding proteins, and storage proteins likely reflects the oxidative stress and inflammatory tumor microenvironment characteristic in CRLM, which can contribute to increased matrix stiffness [60–63]. Furthermore, methionine oxidation is implicated in enhancing TGF-β2-mediated EndMT signaling, consistent with the observed mesenchymal/stromal proteome shift post-chemotherapy [64]. In contrast, cancer cell-dense metastases with scarcer stroma exhibited elevated expression of mitochondrial ribosomal proteins, likely reflecting heightened protein synthesis demands to sustain mitochondrial function and energy production in vital cancer cells. While this shift may be inherently driven by changes in the relative proportions of stromal and cellular components – given that response classification was based on an increased necrotic-to-fibrotic area ratio relative to viable tumor cells – the observed patterns may not merely reflect an abundance of tumor stroma. Alternatively, these findings indicate the involvement of distinct immune cell populations in the highly immunosurveilled and pronouncedly heterogeneous tumor microenvironment of CRLM sustained by tumor-associated macrophages and T- and B-cell infiltrates [14, 47, 65]. Based on the extent of immune infiltration, tumors can be stratified into immunologically “hot” lesions with increased invasion, inflammation and responsiveness to systemic therapy, and “cold” tumors with low immune cell presence, attenuated immunogenicity, and higher grade of chemoresistance [45]. Endothelial anergy, a state marked by inter alias diminished expression of adhesion molecules, is a defining feature of “cold” tumors, impairing immune cell infiltration and thereby creating a microenvironment resistant to immune-mediated attack [45]. The overexpression of a vast variety of immune cell adhesion proteins including multiple subtypes of integrins, leukocyte recruitment proteins, immunoglobulins, chemokines, CD markers and MHC constituents suggests a pronouncedly immune infiltrative and thus “hot” state in responsive metastases [36, 39–41]. Complement factors such as C3 and C5 with their corresponding receptors, anaphylatoxins and chemoattractants can modulate the TME by steering T-cell phenotype, macrophage and monocyte infiltration, and the stabilization of proteins involved in pro-carcinogenic and proliferative pathways in CRC [24, 25, 66–68]. The overlap with stromal and immune invasion markers, alongside EMT signature proteins, upregulation of complement, TGFB-mediated signaling and ECM remodeling may suggest a CMS4 similar nature of the major responsive metastases [28, 29]. While CMS4 tumors are typically more aggressive and prone to early recurrence in primary CRC, recent studies indicate that CMS4-like metastases may exhibit improved prognosis and greater chemosensitivity in the metastatic setting [28–30]. Generally, neoadjuvant chemotherapy has been linked to a mesenchymal shift within molecular subtypes and increase in signatures of EMT and TGF-β [29, 69], with rather CMS4-mesenchymal/stromal subtypes dominating over metabolic an immune signals [29]. Despite these consistent subtype transitions, to the best of our knowledge, no studies have performed clustering analyses stratified by chemotherapy response efficacy [70]. While extracellular matrix (ECM) remodeling constitutes a hallmark of colorectal liver metastases (CRLM), with epithelial-mesenchymal transition (EMT) as a prerequisite for CRC hepatic colonization and formation of conductive tumor microenvironment, our findings provide novel evidence highlighting the critical role of chemotherapy response stratification in clustering analyses, as substantial shifts in viable tumor cell percentage concomitant with increasing ECM proportions may otherwise obscure true ECM remodeling effects and delineation of molecular subtypes in general. The absence of canonical CMS4 markers (e.g., ZEB1, FRMD6, KER, CDX2, HTR2B, integrin-β3) –as primarily endothelial and tumor cell indicators suggests that while the stromal tumor microenvironment dominated by matrisomal markers persists after effective chemotherapy, cellular components have been selectively targeted and depleted [30]. Reduced CMS4 marker expression (e.g., HTR2B, ZEB1, FRMD6) in major responders may reflect loss of viable tumor cells rather than true suppression of mesenchymal programs. These markers are enriched in tumor cells undergoing EMT/mesenchymal transition and are therefore dependent on the presence of viable tumor tissue. In major responders, extensive tumor cell depletion following chemotherapy likely results in lesions dominated by stroma and extracellular matrix, potentially diluting tumor-derived transcriptional signals and leading to lower apparent CMS4 marker expression in bulk analyses. Our analyses thus indicate that the mesenchymal shift in CRLM from other subtypes to CMS4 might be related to effective chemotherapy response characterized by an increase of fibrosis and necrosis in relation to the viable tumor cell count, accompanied by pathway enrichment of ECM remodeling, complement-related factors, and immune infiltration. Distinguishing between chemotherapy-naïve and neoadjuvant-exposed colorectal liver metastases (CRLM) remains essential for describing the molecular landscape of CRLM, and further investigation into clustering by chemotherapy response efficacy is warranted to determine whether CMS4 metastases exhibit superior chemosensitivity, or if effective treatment response itself conceals underlying molecular subtype differences. What is more, in alignment with a serum marker panel used for discrimination of non-metastatic to metastatic CRC, the responsive metastases expressed elevated levels of proteomic complement component C9, A1AG1, alpha-1-antitrypsin, A2GL, yet in contrast a lower expression of FN [71]. The elevated levels of these acute-phase, complement and inflammatory proteins may therefore not merely indicate the aggressive nature of metastasis, but potentially predict chemotherapy response based on their serum presence. Non-invasive molecular profiling could guide choice of treatment regimens in the preoperative context or facilitate surveillance of treatment course. Generally, the absence of significant protein expression changes linked to specific treatment protocols reinforces the robustness of major responsive proteomic profiles as potential indicators of chemotherapy efficacy. The extensive matrisomal alterations and associated post-translational modifications provide a novel basis for linking proteomic changes to measurable viscoelastic properties, such as metastatic stiffness as previously demonstrated by MRE, which could enable noninvasive treatment surveillance [63]. 

Our study has some limitations. While genomic alterations and microRNA expression profiles are similar in CRC and its liver metastases, this genetic similarity only partially extends to the proteomic profile, with differences observed even in metachronous CRLM [51, 72, 73]. This prompted the inclusion of two patients with two-stage resections. A major bias comprises the inclusion of only resectable cases, which excludes patients with unresectable tumors responsive to chemotherapy and thus limits the scope of the study in assessing the broader response of CRLM to adjuvant chemotherapy. Given the limited availability of proteomic data for CRLM, proteomic diversity must be carefully considered when analyzing datasets that encompass CRC broadly, rather than metastasized CRC or CRLM specifically. Although the chemotherapeutic responsiveness of metastases has a profound impact on patient’s prognosis, it is only one contributing factor in a complex prognostic landscape. Our study, which focused on immediate treatment effects, did not explore the correlation to long-term outcomes, limiting a conclusive assessment of their prognostic significance.

While the bulk proteomic approach limits cell-type resolution, our findings provide a systems-level framework for understanding the tumor microenvironment’s role in chemotherapy response and nominate candidate tissue biomarkers for further validation and offering a comprehensive unbiased snapshot of the proteomic composition of tissues. Even if specific cell types cannot be definitively assigned, the convergence of protein markers patterns suggest strong evidence for the wide involvement certain cellular entities, laying a foundation for future studies employing single-cell resolution or spatial techniques in proteomic analyses, and noninvasive therapy surveillance modalities like liquid biopsy.

## Conclusion

Our study revealed significant proteomic changes in response to chemotherapy across histopathological response types in CRLM. Responsive metastases exhibited pronounced changes in the tumor microenvironment, driven by complement-mediated pathways, stromal and immune infiltration, and ECM remodeling. Concurrently, attenuated expression of proteins of mitochondrial ribosomal biogenesis indicated decreased biosynthetic capacity needed for cellular maintenance and growth. The overexpressed proteins in responsive metastases could serve as proteomic biomarker signatures for evaluating treatment efficacy, with longitudinal studies needed to assess their potential prognostic value in the metastatic setting.

## Supplementary Information

Below is the link to the electronic supplementary material.

Supplementary Material 1

Supplementary Material 2

Supplementary Material 3

Supplementary Material 4

Supplementary Material 5

Supplementary Material 6

Supplementary Material 7

Supplementary Material 8

Supplementary Material 9

Supplementary Material 10

## Data Availability

The dataset supporting the conclusions of this article is available in the *PRIDE partner repository* (PXD065812).

## Abbreviations

ANF: Area of necrosis and fibrosis
CAF: Cancer-associated fibroblast
CMS: Consensus molecular subtypes
CRC: Colorectal cancer
CRLM: Colorectal liver metastases
DEP: Differentially expressed proteins
ECM: Extracellular matrix
EMT: Epithelial mesenchymal transition
FC: Fold changes
LC/MS: Liquid Chromatography-Mass Spectrometry
LFQ: Label-free quantification
MR: Major response
NPBCT: Non-platinum-based chemotherapy
NPCT: No preoperative chemotherapy
NR: No response
PBCT: Platinum-based chemotherapy
PCT: Preoperative chemotherapy
PR: Partial response
PTM: Post-translational modification
RB: Rubbia-Brandt
TME: Tumor microenvironment
VTC: Vital tumor cells
