# Supplementary material for: Proteomic profiling of colorectal liver metastases reveals histopathological response-specific molecular signatures of chemotherapy efficacy
Source: J Transl Med. 2026 Mar 5;24:487. doi: 10.1186/s12967-026-07945-1 (PMC13064250; doi:10.1186/s12967-026-07945-1)
Supplement: Supplementary file 1 — Supplementary Material 1 [file 12967_2026_7945_MOESM1_ESM.docx]

**METHODS**

*Methodology of proteomic analysis*

Protein identification was performed using a nano-liquid chromatography electrospray ionization tandem mass spectrometry (LC-MS) approach as previously described by *Wisniewksi* *et al.* (2009).[16] All samples were lyophilized, homogenized, and prepared for LC/MS using the *Filter-Aided Sample Preparation* (FASP) method, involving the injection of 4 µl of eluate into a *nanoUHPLC* system (Dionex UltiMate 3000, Thermo Fisher Scientific, Waltham, MA, USA) coupled with an timsTOF HT flex mass spectrometer (Bruker Daltonics) equipped with a CaptiveSpray nano-electrospray ion source 2 (timsTOF flex HT, Bruker Daltonic GmbH, Bremen, Germany).[16] Spectra libraries were created with pool samples by data in data dependent analyses (DDA), as further described in ***Supplementary Data 1***. Parallel accumulation-serial fragmentation combined with data-independent acquisition (diaPASEF) was performed.[17] Spectra library was used to search against the acquired diaPASEF raw data files via PEAKS studio proteomics search engine (Version 11.5 Bioinformatics Solutions, Waterloo, Canada). Label-free quantification (LFQ) with PEAKS Q was used, whereby PEAKS was allowed to autodetect the reference sample and automatically align the sample runs. To facilitate the export of complete results, the protein significance filter was set to 0, the protein fold change filter to 1, and unique peptide filter to 1 in the export settings. All primary proteomic data was uploaded to the *ProteomeXchange Consortium* via the *PRIDE partner repository* xxx).[18]

**SUPPLEMENTARY**

**Supplementary Data 1: Proteomics Methods**

*Methodology of proteomic analysis*

Spectra library were created (pool sample) by data in data dependent analyses (DDA). Capillary voltage was set to 1600 V, and the spectra range of m/z from 100 to 1700 with an ion-mobility range (1/K0) from 0.85 to 1.30 Vs/cm2. Parallel accumulation–serial fragmentation combined with data-independent acquisition (diaPASEF) was performed by the following settings: PASEF mode with 10 PASEF MS/MS scans. Capillary voltage was set to 1600 V, and the spectra range of m/z from 100 to 1700 with an ion-mobility range (1/K0) from 0.85 to 1.30 Vs/cm2.[1] The ramp and accumulation time were set to 100 ms and the duty cycle close to 100% and a total cycle time of 0.95s. Parameter for MS/SM were set to: Collision energy was ramped linearly from 59 eV at 1/K0 = 1.6 Vs/cm2 to 20 eV at 1/K0 = 0.6 Vs/cm2. Precursors with charge state from 0 to 5 were selected with the target value of 20,000 and intensity threshold of 2500. Any precursors that reached the target value in arbitrary units were dynamically excluded.

*Spectra library*

In order the create a spectra library the UniProt database (Taxonomy: Homo sapiens, Searched Entries: 20,423) was used to search against the acquired DDA raw data files via PEAKS studio proteomics search engine (Version 11.5 Bioinformatics Solutions, Waterloo, Canada) with the following parameters:

De Novo Parameters: Precursor Mass Error Tolerance: 20.00 ppm, Fragment Mass Error Tolerance: 0.05 Da, Enzyme: Trypsin, Maximum Variable PTM per Peptide: 2, Fixed Modifications: Carbamidomethylation (+57.02), Variable Modifications: Oxidation (M) (+15.99).

*Database Search Parameters:* Precursor Mass Error Tolerance: 20.00 ppm, Fragment Mass Error Tolerance: 0.05 Da, Enzyme: Trypsin, Maximum Missed Cleavage: 2, Digestion Mode: Semi-specific, Peptide Length Range: 6 - 45, Maximum Variable PTM per Peptide: 2, Fixed Modifications: Carbamidomethylation (+57.02), Variable Modifications: Oxidation (M) (+15.99), Peptide FDR: 1.0%, Proteins -10LgP ≥ 15.0,

Proteins Unique Peptides ≥ 1, De Novo Only ALC ≥ 50.0%.[2]

*Library search and Label-free quantification*

Spectra library (Taxonomy: Homo sapiens, Searched Entries: 20,423) was used to search against the acquired diaPASEF raw data files via PEAKS studio proteomics search engine (Version 11.5 Bioinformatics Solutions, Waterloo, Canada). DIA DB Search Parameters were applied as follows: Precursor Mass Error Tolerance: 20.00 ppm, Fragment, Mass Error Tolerance: 0.05 Da, CCS Error Tolerance: 0.05 Max Missed Cleavage: 1, Digest Mode: Semi-Specific, Max Variable PTM per Peptide: Fixed Modifications: Carbamidomethylation (+57.02), Variable Modifications: Oxidation (M) (+15.99), Taxonomy: Homo sapiens (human), Peptide Length between: 7,30, Precursor M/Z between: 300,1800, Fragment M/Z between: 200,1800, Precursor Charge between 1-4, Peptide FDR: 1.0% Proteins -10LgP >= 15.0 Proteins Unique Peptides >= 1.

**References**

[1] F. Meier, A.D. Brunner, M. Frank, A. Ha, I. Bludau, E. Voytik, S. Kaspar-Schoenefeld, M. Lubeck, O. Raether, N. Bache, R. Aebersold, B.C. Collins, H.L. Rost, M. Mann, diaPASEF: parallel accumulation-serial fragmentation combined with data-independent acquisition, Nat Methods, 17 (2020) 1229-1236.

[2] C. UniProt, UniProt: the Universal Protein Knowledgebase in 2025, Nucleic Acids Res, 53 (2025) D609-D617.
