# Supplementary material for: Proteomic profiling of colorectal liver metastases reveals histopathological response-specific molecular signatures of chemotherapy efficacy
Source: J Transl Med. 2026 Mar 5;24:487. doi: 10.1186/s12967-026-07945-1 (PMC13064250; doi:10.1186/s12967-026-07945-1)

**Supplementary Data 3: PCA with individual patients**

3.1 Whole proteome


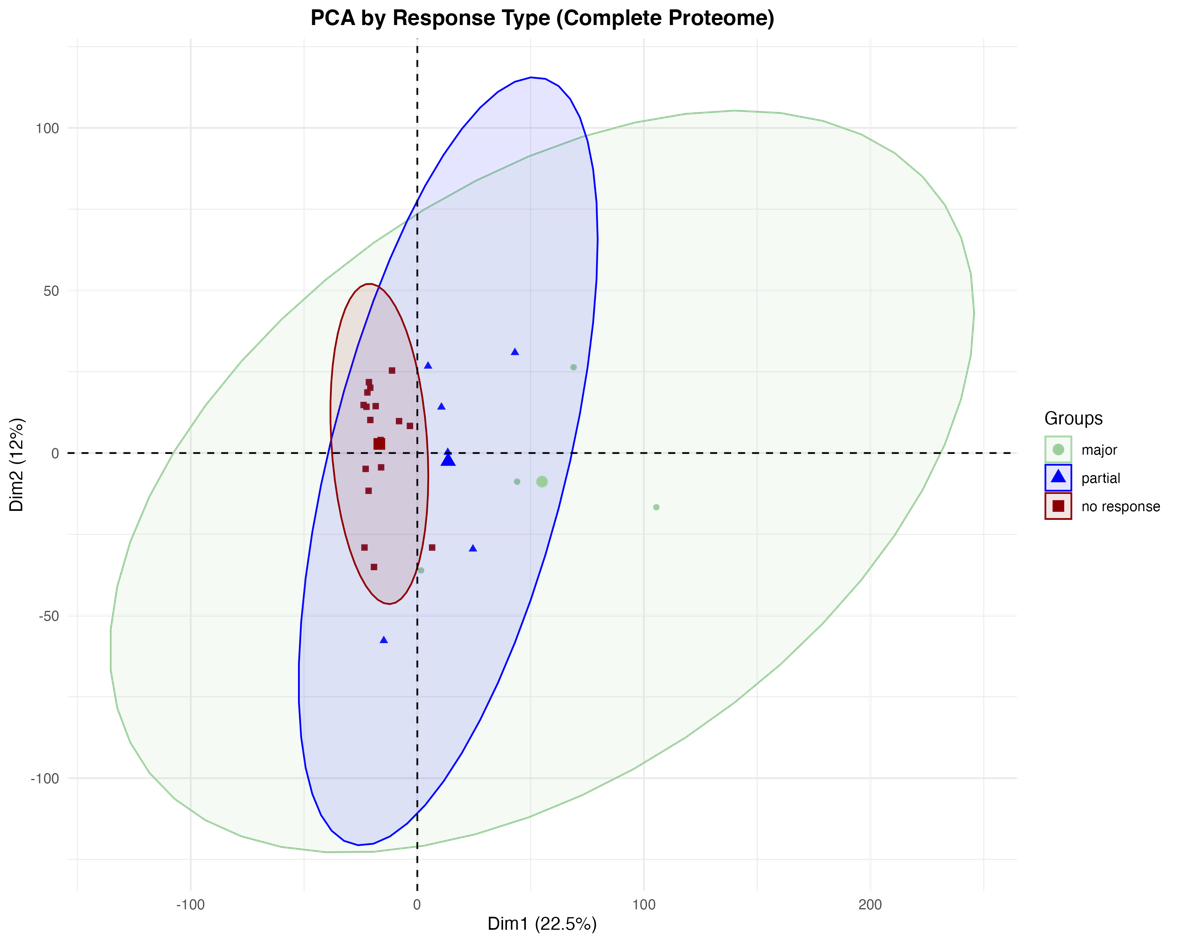


3.2 Differentially expressed proteins


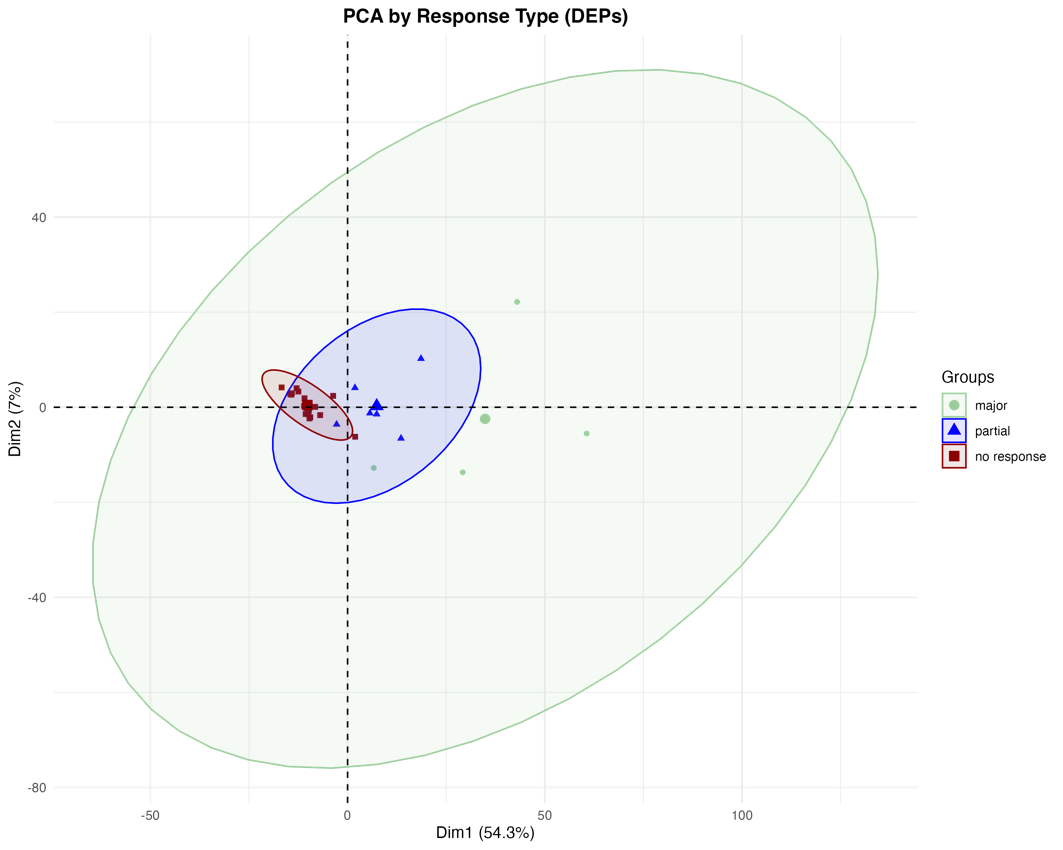

Supplement: Supplementary file 6 — Supplementary Material 6 [file 12967_2026_7945_MOESM6_ESM.docx]
